# Elusive *Enterobacter cloacae* causing pacemaker endocarditis

**DOI:** 10.1016/j.idcr.2021.e01149

**Published:** 2021-05-07

**Authors:** Naji Maaliki, Jorge Verdecia, Malleswari Ravi

**Affiliations:** a Department of Internal Medicine, University of Florida COM-Jacksonville, 4th Floor, 655 8th W 8th Street, Jacksonville, FL, USA; b Department of Infectious Diseases, University of Florida COM-Jacksonville, 655 8th W 8th Street, Jacksonville, FL, USA

**Keywords:** Endocarditis, *Enterobacter cloacae*, Non-HACEK gram-negative rods, Cardiac implantable electronic device endocarditis, Pacemaker endocarditis

## Abstract

An 80-year-old patient was admitted for fever, chills, and chest wall pain. He had a past medical history significant for heart failure with a cardiac resynchronization therapy pacemaker implantation. Extensive workup revealed *Enterobacter cloacae* endocarditis of the pacemaker leads and the mitral valve, a rare etiology with an unidentified source in our patient. He was managed with a rather unconventional method which proved to be successful. This case sheds light on non-HACEK (other than *Haemophilus* spp., *Aggregatibacter actinomycetemcomitans*, *Cardiobacterium hominis*, *Eikenella corrodens*, or *Kingella* spp). gram-negative organisms, and particularly *E. cloacae*, as uncommon causes of endocarditis with elevated mortality, and discusses potential treatment modalities.

## Introduction

Infectious endocarditis (IE) is an infection of the endocardial surface which is detrimental if improperly treated. Non-HACEK gram-negative bacilli rarely cause IE, and very few cases have reported *Enterobacter cloacae* endocarditis. Due to its rare occurrence, it has become a diagnostic and treatment challenge. We present a case of an 80-year-old patient who was found to have IE from *E. cloacae*, infecting both the mitral valve and his cardiac implantable electric device (CIED).

## Case presentation

**Fig. 1:**
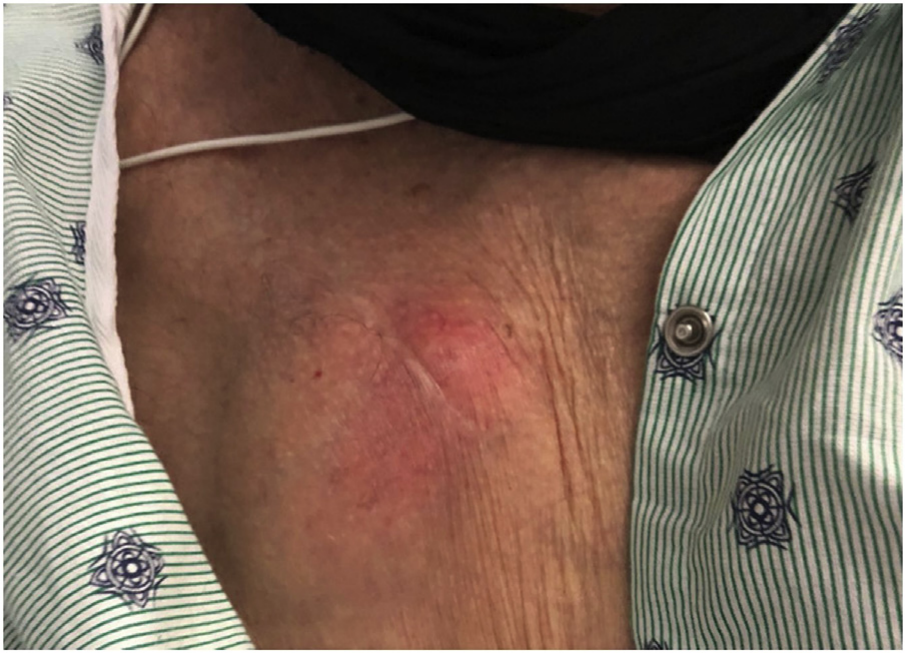
Erythema and swelling around CRT-P insertion site.

**Fig. 2:**
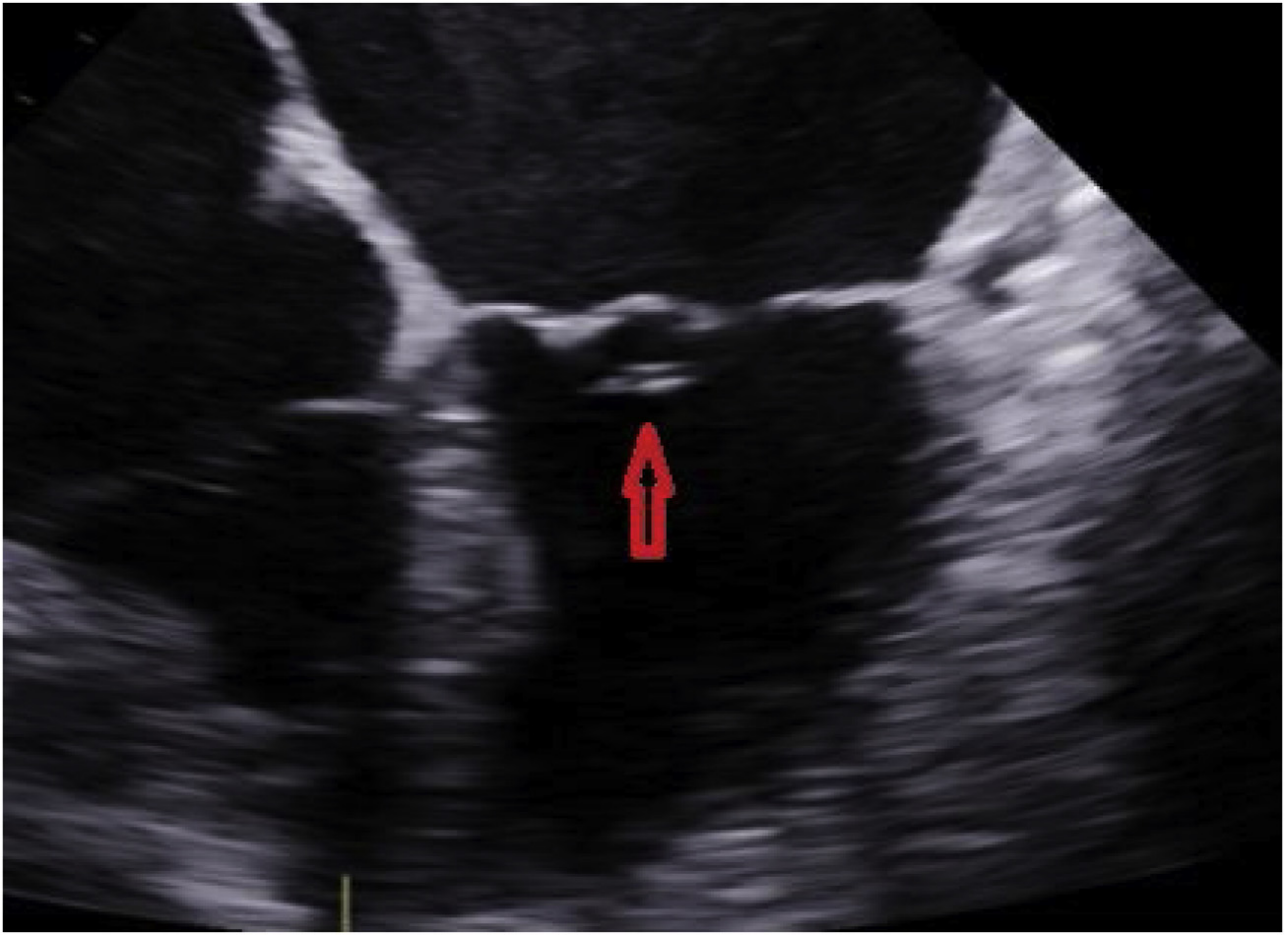
TEE revealing vegetation at the anterior leaflet of the mitral valve.

**Fig. 3:**
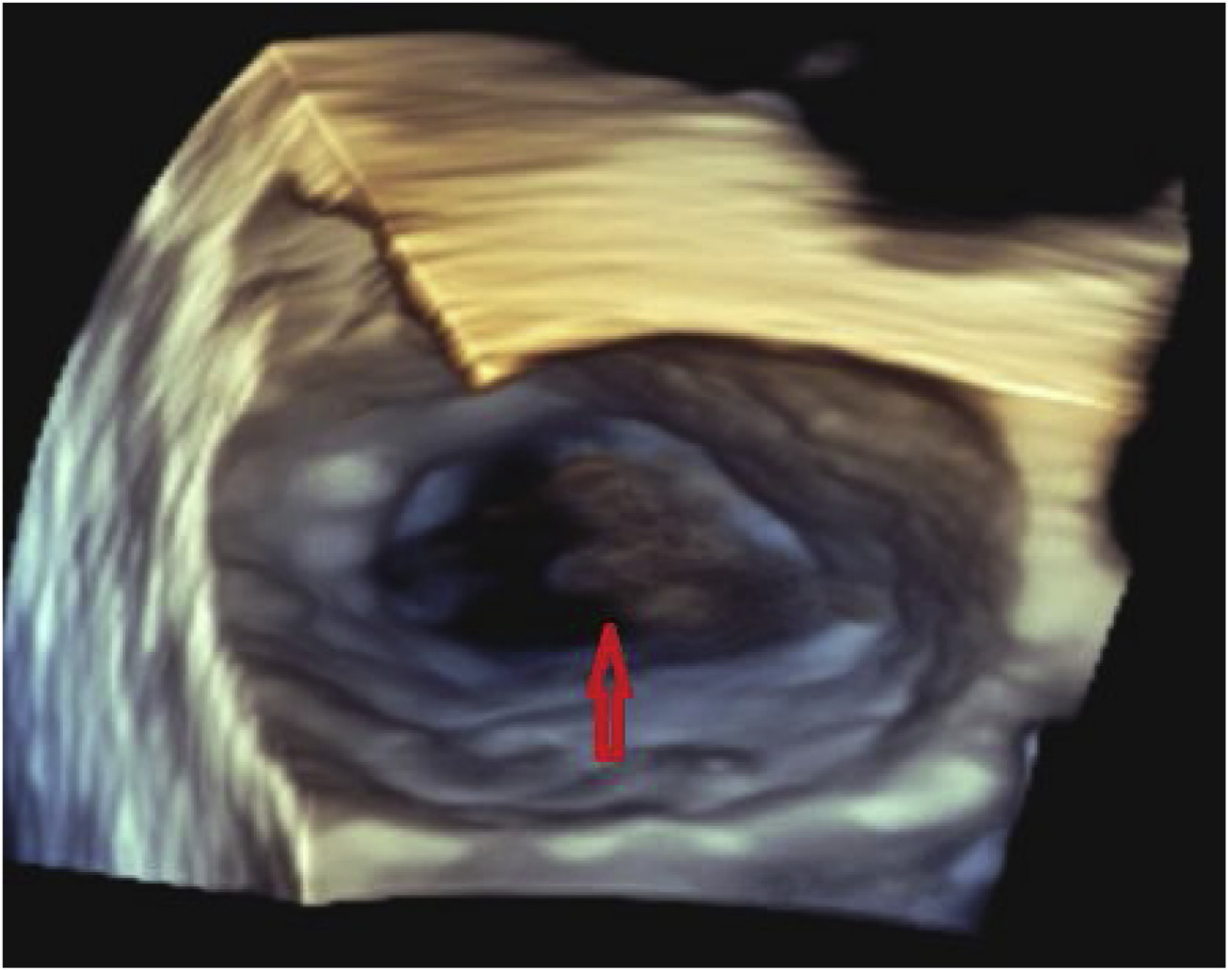
3D TEE demonstrating an anterior vegetation on the Mitral valve.

**Fig. 4:**
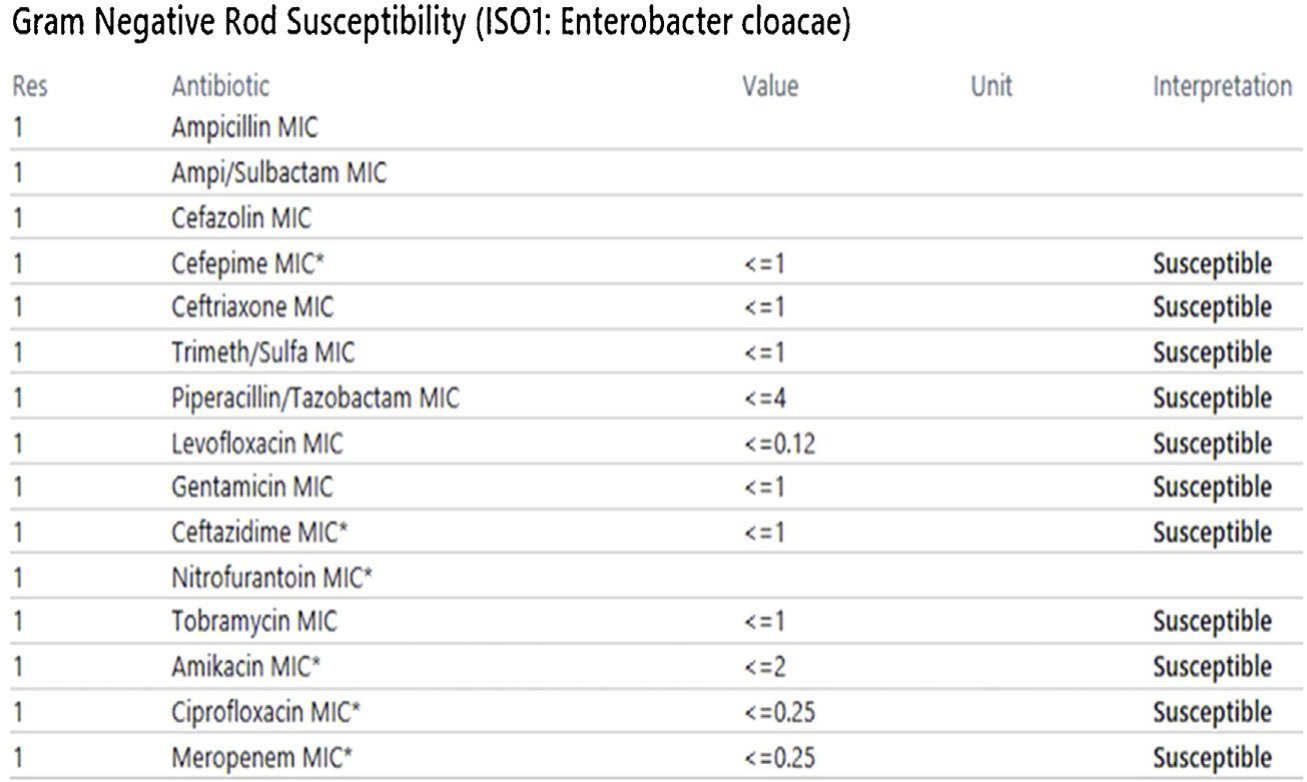
Respective MIC per Antimicrobial for *E. cloacae*.

An 80-year-old male presented to our hospital with fever and chest wall pain. Medical history of hypertension, atrial fibrillation, and heart failure with recovered ejection fraction after a cardiac resynchronization therapy pacemaker (CRT-P) implantation. He complained of intermittent fevers, chills, and lethargy for the past month, associated with a 3-day history of pain and swelling at the CRT-P implant site. Physical exam showed erythema, swelling, and tenderness to palpation at the left chest surrounding the pacemaker insertion site (Fig. 1). Workup revealed an elevated white blood cell count and gram-negative bacteremia, later confirmed to be *E. cloacae*. Treatment started with both vancomycin and cefepime due to concern for a gram-positive pacemaker infection independent of the known *Enterobacter*. Chest x-ray displayed a well-positioned CRT-P with proper lead placement, and an electrocardiogram confirmed a paced rhythm. Transthoracic and transesophageal echocardiography demonstrated vegetation on the anterior leaflet of the mitral valve suggestive of endocarditis, but no vegetation on the device leads (Fig. 2, Fig. 3); nonetheless, the electrophysiology team performed a percutaneous CRT-P extraction on hospital day 7. The pacemaker pocket and lead cultures grew *E. cloacae* as well. Treatment was narrowed to cefepime monotherapy targeting pacemaker and mitral valve endocarditis (Fig. 4). A new CRT-P was placed 14 days after the extraction. Further investigation with computed tomography of the abdomen and pelvis with contrast, and urinalysis, did not divulge the source of infection. The patient reported dysuria nine weeks earlier that was empirically treated with trimethoprim-sulfamethoxazole (Bactrim) for ten days without urinalysis. Three weeks later, he received doxycycline for ten days for fevers and chills. One month before admission, he presented to urology for the recurrence of this fever and chills. The evaluation had revealed a non-tender prostate, unremarkable urinalysis, and a PSA of 1.1, thus ruling out prostatitis. Three days before this admission, he had a routine colonoscopy with the removal of a few polyps. Eventually, the patient was safely discharged on cefepime therapy to complete six weeks. On the follow-up visit, he continued to do well with significant improvement of symptoms and no re-hospitalizations.

## Discussion

Infective endocarditis (IE) is the infection of the heart's endocardial lining, such as the heart valves and intracardiac devices [1]. It is a potentially lethal disease mostly caused by bacteria and less commonly fungi. Risk factors include pre-existing valve disease, prosthetic valves, implantable devices, intravenous drug use, and immunocompromised status. Diagnosis is through the Modified Duke Criteria, which includes major criteria of two separate positive blood cultures of typical microorganisms consistent with IE and visual evidence of endocardial involvement through echocardiography [1]. Management involves a multi-disciplinary approach, and treatment includes an extended course of intravenous antibiotics and surgical consideration in some instances [2].

Gram-negative bacilli are an uncommon cause of endocarditis, with most cases being due to gram-positive cocci [3]. IE caused by non-HACEK gram-negative bacilli is especially rare, amounting to 1.8 %–3.9 % of IE cases in recent meta-analyses [4,5]. Specifically, *E. cloacae* endocarditis has seldom been reported, with as few as 14 cases in a recent review, only 2 of which were associated with CIED [6].

*E. cloacae* is a gram-negative, facultative anaerobic bacillus that is part of the gut flora. It is mostly associated with nosocomial bacteremia, urinary tract infections, pneumonia, and rarely endocarditis [7]. Contrary to the usual association of right-sided endocarditis with gram-negative rods, the *Enterobacter* species has been most associated with mitral valve infections [6]. Common predispositions include prior valve disease, prosthetic valves, CIEDs, and prolonged vascular access [8]. Due to the highly uncommon incidence of endocarditis with *E. cloacae*, or the non-HACEK gram-negative bacilli in general, its management has continued to pose a conundrum. Current guidelines endorsed by the Infectious Diseases Society of America and the American Heart Association recommend dual antibiotic therapy and early cardiothoracic surgical consultation in non-HACEK endocarditis cases [8,9]. The suggested therapy is a fourth-generation cephalosporin or carbapenem, along with an aminoglycoside or quinolone for a total of 6 weeks [6,8,9]. Due to the Amp C inducible beta-lactamases expression associated with non-HACEK gram-negative rods that provide resistance to multiple beta-lactam antibiotics, consultation with an Infectious Disease specialist is merited as additional laboratory screening and in-vitro testing would be required [2,10]. In cases of CIED endocarditis, urgent removal of the device is warranted [11]. While involving the cardiothoracic surgery team seems to be universally supported, reports vary regarding outcomes as some cite increased mortality with surgery (44 % with surgery vs. 30 % without surgery) [12]. Regardless of the multi-disciplinary approach, endocarditis due to non-HACEK gram negative-rods and *E. cloacae* has seen an elevated mortality rate of around 24 % and 42.9 %, respectively [2,6].

In our case, the source of bacteremia remained unconfirmed. Potentially, it could have been from the colonoscopy allowing for gut-flora translocation, although his fever and chills episodes started before colonoscopy. Our management differed from convention as treatment focused on a 6-week course of cefepime monotherapy, which successfully cleared the infection. We chose this method because the organism was susceptible with a low MIC (<2), repeat blood cultures obtained 48 h after admission became sterile, the patient remained afebrile on cefepime, and the CRT-P was removed along with an incision and drainage of the pocket. This may be an alternative to dual therapy, as this strategy may help decrease antibiotic usage, resistance, and drug-related adverse effects. Aminoglycosides are well-associated with renal toxicity, leading to elevated mortality in cardiac patients due to subsequent electrolyte derangements and toxin accumulation [12]. Quinolones may lead to neurotoxicity, QT-interval prolongation, gastrointestinal upset, and musculoskeletal injury, which become more apparent in the elderly population. As quinolones are renally-excreted, they may be especially detrimental to elderly patients with renal and cardiac disease [13].

Previously, carbapenems were considered the most stable drug against Amp C beta-lactamases [14]. Because widespread use of carbapenems could exacerbate the multidrug-resistant gram-negative organism crisis, studies have investigated other options and found that cefepime is an exception to the recommendation to avoid all cephalosporin therapy for invasive infections caused by these organisms [[15], [16], [17]]. The outcome was favorable with cefepime in treating invasive Amp-C producing infections, especially when the MIC was low, when adequate source control was achieved, and with 8-h dosing. [16,17]. Cefepime has a net neutral charge that gives the advantage of rapidly penetrating bacterial outer membranes, allowing it to readily reach its target compared with other cephalosporins with a net positive charge such as ceftriaxone. Furthermore, cefepime has reduced affinity for B-lactamases and a poor inducer of AmpC B-lactamases [[15], [16], [17]].

## Conclusion

Little is still known about *Enterobacter cloacae* endocarditis, owing to its rare incidence and high mortality. Our successful treatment provides hope for better outcomes with *Enterobacter* endocarditis with 4th generation cephalosporin monotherapy, reducing multiple medications, side-effects, and resistance. As more cases are recognized, we may determine the optimal management strategy for such patients, improving survival and minimizing excess interventions.

## Sources of funding

This research did not receive any specific grant from funding agencies in the public, commercial, or not-for-profit sectors.

## Ethical approval

No violations of patients privacy was done.

## Consent

Written informed consent was obtained from the patient for publication of this case report and accompanying images.

## Author contributions

Naji Maaliki: Acquisition of data, Drafting of the manuscript, Critical Revision of the manuscript.

Jorge Verdecia: Acquisition of data, Drafting of the manuscript, Critical Revision of the manuscript.

Malleswari Ravi: Acquisition of data, Drafting of the manuscript, Critical Revision of the manuscript.

## Declaration of Competing Interest

The authors report no declarations of interest. There was no funding for this manuscript.

## References

[1] Klein M., Wang A. (2016). Infective endocarditis. J Intensive Care Med.

[2] Baddour L.M., Wilson W.R., Bayer A.S., Fowler V.J., Tleyjeh I.M., Rybak M.J. (2015). Infective endocarditis in adults: diagnosis, antimicrobial therapy, and management of complications: a scientific statement for healthcare professionals from the American Heart Association. Circulation.

[3] Chambers S.T., Murdoch D., Morris A., Holland D., Pappas P., Almela M. (2013). HACEK infective endocarditis: characteristics and outcomes from a large, multi-national cohort. PLoS One.

[4] Falcone M., Tiseo G., Durante-Mangoni E., Ravasio V., Barbaro F., Ursi M.P. (2018). Risk factors and outcomes of endocarditis due to non-HACEK gram-negative Bacilli: data from the prospective multicenter Italian endocarditis study cohort. Antimicrob Agents Chemother.

[5] Morpeth S., Murdoch D., Cabell C.H., Karchmer A.W., Pappas P., Levine D. (2007). Non-HACEK gram-negative bacillus endocarditis. Ann Intern Med.

[6] Wengrofsky P., Soleiman A., Benyaminov F., Oleszak F., Salciccioli L., McFarlane S.I. (2019). Enterobacter cloacae device endocarditis: case report, scoping study, and guidelines review. Cardiol Vasc Res (Wilmington).

[7] Mezzatesta M.L., Gona F., Stefani S. (2012). Enterobacter cloacae complex: clinical impact and emerging antibiotic resistance. Future Microbiol.

[8] Karasahin O., Yildiz Z., Unal O., Arslan U. (2018). A rare cause of healthcare-associated infective endocarditis: Enterobacter cloacae. IDCases.

[9] Yoshino Y., Okugawa S., Kimura S., Oleszak F., Salciccioli L., McFarlane S.I. (2015). Infective endocarditis due to Enterobacter cloacae resistant to third- and fourth-generation cephalosporins. J Microbiol Immunol Infect.

[10] Wargo K.A., Edwards J.D. (2014). Aminoglycoside-induced nephrotoxicity. J Pharm Pract.

[11] De Silva K., Fife A., Murgatroyd F., Gall N. (2009). Pacemaker endocarditis: an important clinical entity. BMJ Case Rep.

[12] Moon J., Smith T., Sahud A.G., Bhanot N. (2012). An unusual etiology of infective endocarditis: Enterobacter cloacae. J Infect Chemother.

[13] Norrby S.R. (1991). Side-effects of quinolones: comparisons between quinolones and other antibiotics. Eur J Clin Microbiol Infect Dis.

[14] Rodríguez-Baño J., Gutiérrez-Gutiérrez B., Machuca I., Pascual A. (2018). Treatment of infections caused by extended-spectrum-beta-lactamase-, AmpC-, and carbapenemase-producing Enterobacteriaceae. Clin Microbiol Rev.

[15] Tamma P.D., Girdwood S.C., Gopaul R., Tekle T., Roberts A.A., Harris A.D. (2013). The use of cefepime for treating AmpC β-lactamase-producing Enterobacteriaceae. Clin Infect Dis.

[16] Siedner M.J., Galar A., Guzmán-Suarez B.B., Kubiak D.W., Baghdady N., Ferraro M.J. (2014). Cefepime vs other antibacterial agents for the treatment of Enterobacter species bacteremia. Clin Infect Dis.

[17] Tamma P.D., Doi Y., Bonomo R.A., Johnson J.K., Simner P.J., Antibacterial Resistance Leadership Group (2019). A primer on AmpC β-lactamases: necessary knowledge for an increasingly multidrug-resistant world. Clin Infect Dis.

